# Gastric Pouch Mixed Adenoneuroendocrine Carcinoma With a Mixed Adenocarcinoma Component After Roux-en-Y Gastric Bypass

**DOI:** 10.1177/2324709617740908

**Published:** 2017-11-13

**Authors:** Ricardo G. Pastorello, Mariana Petaccia de Macedo, Wilson Luiz da Costa Junior, Maria Dirlei F. S. Begnami

**Affiliations:** 1 A.C. Camargo Cancer Center, São Paulo, Brazil

**Keywords:** gastric cancer, mixed tumor, MANEC, mixed adenoneuroendocrine carcinomas, neuroendocrine tumors, gastric bypass

## Abstract

The Roux-en-Y gastric bypass is one of the most common procedures currently performed for surgical treatment of patients with severe obesity. Gastric cancer after bariatric surgery is not common, with most of them arising in the excluded stomach. Gastric mixed adenoneuroendocrine carcinomas are a rare type of stomach malignancy, composed of both adenocarcinoma and neuroendocrine tumor-cell components, with the latter comprising at least 30% of the whole neoplasm. In this article, we report a unique case of a mixed adenoneuroendocrine carcinoma with a mixed adenocarcinoma (tubular and poorly cohesive) component arising in the gastric pouch of a patient who underwent previous Roux-en-Y gastric bypass for glycemic control. Since stomach cancer is not usual in patients who have formerly undergone bariatric surgery and symptoms tend to be nonspecific, such diagnosis is often rendered at an advanced stage. Full assessment of these patients when presenting such vague symptoms is critical for an early cancer diagnosis.

## Introduction

In spite of a steadily declining incidence over the past 5 decades, gastric cancer is the fifth most common malignancy worldwide. The incidence of these neoplasms after bariatric surgery, however, is uncommon, with few cases reported in the literature, most of them arising in the excluded stomach. Gastric mixed adenoneuroendocrine carcinomas (MANECs) are a rare type of gastric malignancy, composed of both adenocarcinoma and neuroendocrine tumor-cell components. In this article, we report a unique case of a MANEC with a mixed adenocarcinoma component arising in the gastric pouch of a patient who underwent previous gastric bypass for glycemic control.

## Case Report

A 61-year-old male, former cigarette smoker, with diabetes mellitus and a past medical history of Fobi-Capella Roux-en-Y gastric bypass for glycemic control in an outside institution, developed an incisional hernia 2 months after the operation. Nineteen months after the procedure, the patient was then submitted to an upper gastrointestinal endoscopy in our service for further evaluation of the hernia before repair surgery. At the time, he was asymptomatic with no significant findings in the clinical examination besides a 10.0-cm hernia over the abdominal midline incision. The endoscopic exam revealed a vegetating, friable lesion in the gastric pouch, near the gastroesophageal junction, measuring approximately 2.0 cm in the greatest diameter. A biopsy of the lesion was performed.

The hematoxylin-eosin slides of the endoscopic biopsy revealed a neuroendocrine neoplasm. Ancillary immunohistochemistry (IHC) studies showed positivity for cytokeratin pool AE1/AE3, cytokeratins 8/18, CD56, synaptophysin, chromoganin A, and Ki-67 index of 30% of the neoplastic cells. Based on those findings, a diagnosis of neuroendocrine carcinoma according to the 2010 World Health Organization (WHO) classification^1^ was rendered. For staging purposes, computed tomography and an endoscopic ultrasound were performed, which showed a paraesophageal adenomegaly and no signs of invasion of the muscularis propria (cT1 cN1 cM0—TNM 8th edition).^2^ The patient was then submitted to total gastrectomy with D2 lymph node (LN) dissection and hernia repair.

**Figure 1.**
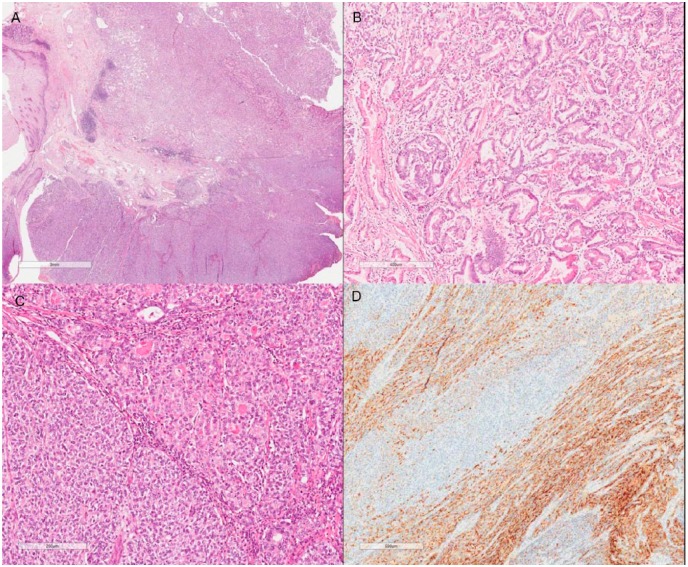
Mixed adenoneuroendocrine carcinoma (A; hematoxylin-eosin [HE], original magnification 100×) constituted by an adenocarcinoma component (B; HE, original magnification 200×) in association with a neuroendocrine carcinoma (C; HE, original magnification 200×). Chromoganin immunohistochemical stain is positive in the neuroendocrine component (D; original magnification 200×).

**Figure 2.**
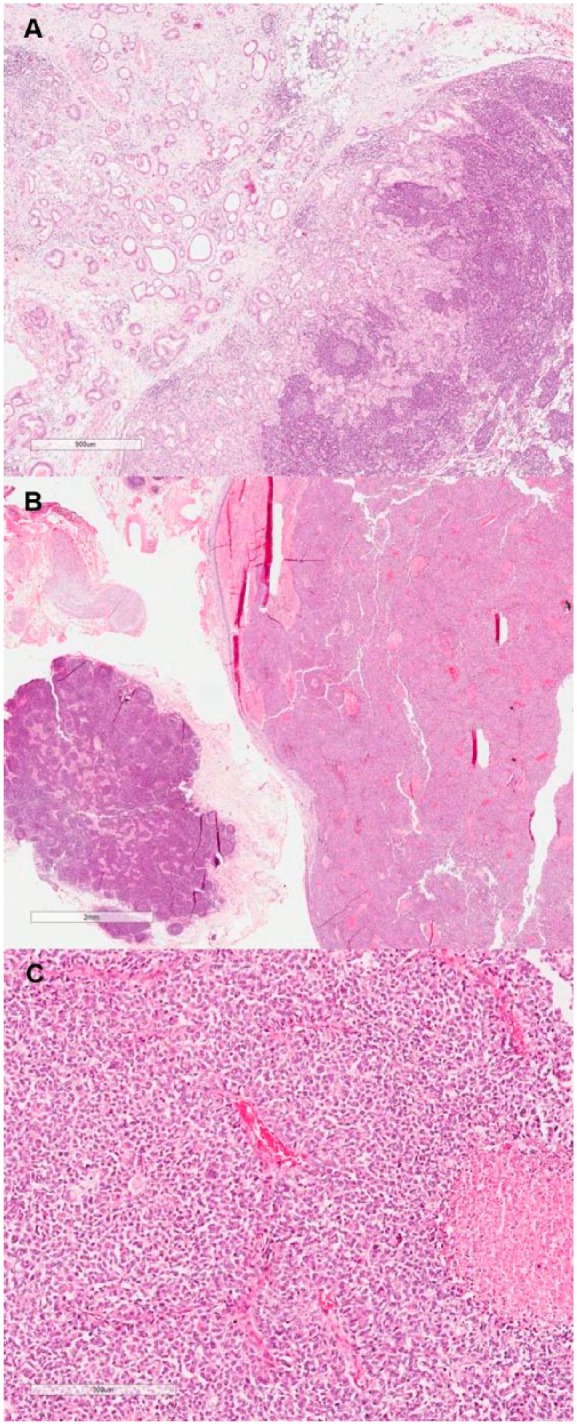
Lymph node metastasis of the adenocarcinoma component (A; hematoxylin-eosin [HE], original magnification 100×) in contrast to another lymph node infiltrated by the neuroendocrine component (B; HE, original magnification 100×). High-power view of the neuroendocrine carcinoma metastatic component (C; HE, original magnification 200×).

Grossly, the gastrectomy product showed a 2.0 × 1.5 cm elevated, vegetating lesion. Microscopic examination revealed a MANEC (WHO 2010^1^), constituted by a mixed adenocarcinoma (tubular and poorly cohesive) in association with a neuroendocrine carcinoma (Figure 1), constituting 40% and 60% of the lesion, respectively. The tumor infiltrated on the submucosa, and lymphovascular invasion was detected. The surgical margins were free of tumor cells. The LN dissection revealed 24 LNs, with 2 of them from the lesser curvature compromised by the neoplasia. Surprisingly, one of the LNs was infiltrated by a pure neuroendocrine carcinoma whereas the other was compromised by a pure tubular adenocarcinoma component (Figure 2). The IHC panel findings from the surgical specimen were similar to the biopsy findings. The hernia sac showed no histologic abnormalities.

Laboratory and imaging postoperative examinations did not reveal residual or metastatic disease. A 12-cycle course of adjuvant chemotherapy including folinic acid, fluorouracil, and oxaliplatin (FOLFOX) was initiated, started at nearly 3 months after the surgery, and the patient tolerated it well.

Almost 3 years after the gastrectomy, the patient had to undergo a small bowel resection due to an acute obstruction caused by adhesions. Three years later, he presented with an expansive retroperitoneal formation on imaging follow-up exams. A computed tomography–guided biopsy of this lesion revealed infiltration of nodal and soft tissues by a pure adenocarcinoma, confirming the recurrence of the disease, this time as a single-component neoplasm. At the time of writing, the patient is now about to start a new course of systemic treatment.

## Discussion

Gastric cancer is the fifth most common malignancy worldwide. In spite of a steadily declining incidence over the past 5 decades, nearly 1 million new cases of these cancers were estimated to have happened in 2012, with more than 70% of cases occurring in developing countries.^3,4^

High body mass index and obesity have been associated with increased risk for cancer.^5^ Surgery is a treatment option in morbidly obese patients known for effective weight loss and beneficial long-term results. The Roux-en-Y gastric bypass (RYGBP) is one of the most common procedures currently performed for surgical treatment of severe obesity.^6^ The incidence of gastric cancer after bariatric surgery is rare, with few cases reported in the literature,^7^ most of adenocarcinomas arising in the excluded stomach,^8^ in contrast to our patient’s tumor location in the gastric pouch.^6,9-14^

Gastric MANEC is a rare type of gastric cancer.^15^ As defined by the 2010 WHO classification, MANECs are composed of both adenocarcinoma and neuroendocrine tumor-cell components, with the latter comprising at least 30% of the whole neoplasm.^1^ According to the WHO, a minor (<30%) neuroendocrine component can often be present within gastric adenocarcinomas and these tumors should not be classified as MANECS. As stated by Stojsic et al,^16^ these neoplasms should be regarded as adenocarcinomas with focal neuroendocrine differentiation. Traditionally, mixed epithelial and endocrine cell type tumors were first classified into 3 groups (composite, collision, and amphicrine tumors) based on the 2 components’ relation and distribution.^17^ The use of different names in the literature to describe these neoplasms led to confusion among clinicians and pathologists.^18^ In 2000, the WHO classification defined them as mixed exocrine-endocrine carcinomas, and 10 years later, in the current classification, the WHO renamed them as MANECs.^1,19^

Due to its rarity, scarce specific epidemiological data are available for MANECs in the literature. Furthermore, few aspects are known about their etiology. It is likely that these neoplasms may originate from either the simultaneous proliferation of distinct lines of cells or the proliferation of stem cells able to differentiate into multiple cell lineages.^17,20,21^

Mixed adenoneuroendocrine carcinomas may occur at any site in the stomach.^22-24^ Although there are rare descriptions of these tumors arising after gastric surgery,^25,26^ there have been no reports in the English literature so far of gastric MANECs occurring after RYGBP, particularly in the gastric pouch, as depicted herein.

Clinically, no specific symptoms differing from conventional gastric malignancies have been described to be related to MANECs,^27^ and some authors indicate that their clinical behavior depends mostly on the neuroendocrine component.^28,29^ In patients who underwent gastric bypass, stomach cancers tend to present with vague manifestations, including abdominal discomfort, bloating, nausea, and weight loss. Such nonspecific symptoms may be easily confused with the ones secondary to the bypass itself. As a result, a malignancy diagnosis is often rendered at an advanced stage. Full assessment of these patients when presenting analogous symptoms is critical for an early cancer diagnosis.^7,8,30^

On gross examination, MANECs usually present the same features as conventional gastric cancer.^1^ Microscopically, the neuroendocrine component is normally composed of a high-grade neuroendocrine carcinoma, and uncommonly a well-differentiated neuroendocrine tumor.^22,23^ The non-neuroendocrine component is commonly an adenocarcinoma with variable degrees of differentiation.^29,31^ On exceedingly rare reports in the literature, as illustrated by our case, the exocrine component is formed by a mixed adenocarcinoma.^32^ As with pure neuroendocrine tumors, MANECs display positivity for neuroendocrine markers on IHC studies restricted to the neuroendocrine component, with expression of carcinoembryonic antigen in some of these cases as well.^15,22^ There are no data supporting differences in outcome regarding the proportion of each adenocarcinoma and neuroendocrine component of the neoplasm, and so there is no specific guideline in the literature suggesting the necessity to specify the proportion of each neoplasm component in the pathology report of an MANEC.

Genetic studies on gastric MANECs are scarce. Evidence on these indicates a rather higher frequency of chromosomal abnormalities in the neuroendocrine carcinoma in comparison to the adenocarcinoma component. Nonetheless, shared loss of heterozygosity at specific chromosomes proposed a close genetic relation and a potential multistep evolution from a common precursor lesion.^23,33^

The prognosis for patients with gastric MANECs is usually poor.^15^ The 5-year survival rate is lower for these patients than for those with conventional stomach adenocarcinomas, and the neuroendocrine component may have a critical role in the prognosis.^34^ A good prognosis of gastric MANEC is rare and normally restricted to tumors detected in their early stages.^35^

There is no optimal treatment strategy to date in the management of MANECs.^18^ Some authors suggest that the most aggressive component should be taken into account when considering the best treatment option.^36-38^ Surgical resection is mostly indicated and is usually followed by adjuvant therapy.^39,40^

Previous studies have shown that in MANECs the adenocarcinoma component was mostly located in the mucosa and submucosa, whereas the neuroendocrine component is in the deeper portions of the gastric wall,^23,41,42^ making a definitive diagnosis of MANEC difficult on preoperative endoscopic biopsy.

In conclusion, we report a unique case gastric cancer arising in a patient who underwent previous Roux-en-Y gastric bypass for glycemic control with 3 very unusual presentations: being localized in the gastric pouch instead of the excluded stomach and histologically present as an MANEC with the additional finding of harboring a mixed adenocarcinoma component. Though gastric cancer is not usual in patients who have formerly undergone bariatric surgery, it is crucial to monitor this population for the development of such malignancy, performing a full and thorough medical evaluation if they develop vague symptoms.
